# Predicting Treatment Cost for Bacterial Diarrhoeaat a Regional Hospital in Thailand*

**DOI:** 10.3329/jhpn.v26i4.1886

**Published:** 2008-12

**Authors:** Arthorn Riewpaiboon, Kwanduen Intraprakan, Sukit Phoungkatesunthorn

**Affiliations:** 1 Faculty of Pharmacy, Mahidol University, Bangkok 10400, Thailand; 2 Saraburi Hospital, Saraburi, Thailand

**Keywords:** Bacteria, Cost-function analysis, Cost and cost analysis, Diarrhoea, Health expenditure, Healthcare cost, Retrospective studies, Thailand

## Abstract

The aim of this study was to estimate the treatment cost and formulate a cost-function for bacterial diarrhoea among patients in a Thai regional hospital. This study was an incidence-based cost-of-illness analysis from a hospital perspective, employing a micro-costing approach. It covered new episodes of both outpatients and inpatients who were diagnosed to have bacterial diarrhoea (ICD-10 code A00-A05) and who received treatment during 1 October 2000–31 July 2003. Retrospective data were collected from medical records of the hospital. The study covered 384 episodes, and the mean age of patients was 24 years. The average treatment costs (at 2002 prices; US$ 1=approximately 40 Thai baht) were US$ 11.29, 76.78, and 44.72 per outpatient episode, inpatient episode, and outpatient/inpatient combined episode respectively. Furthermore, the positive significant predictor variables were: inpatient care, other *Salmonella*-associated infections, shigellosis, other bacterial intestinal infections, and the health insurance scheme. The fitted model was able to predict greater than 80% of the treatment cost. The estimation of simulated patients demonstrated a wide range of costs, from US$ 10 per episode to US$ 163 per episode. Overall, hospital administrators can apply these results in cost-containment interventions.

## INTRODUCTION

Thailand is a Southeast Asian country with a population of 62.4 million in 2005. Acute diarrhoea is one of the most prevalent diseases in Thailand, and morbidity rate pertaining to this disease has been ranked the highest in the country since 1985. Results of an epidemiological study in four hospitals in 2005 indicated that most diarrhoea patients were children aged less than five years (35.2%). *Salmonella* spp. (39.9%), *Vibrio parahaemolyticus* (33.1%), *Escherichia coli* (18.0%), *Shigella* spp. (2.5%), and *Vibrio cholerae* El Tor Inaba (1.8%) were mainly responsible for the disease (1). The number of inpatients at government hospitals (80.5% of the total hospital-beds in the country in 2004) was 11,323.8 per 100,000 in 2004. Meanwhile, the numbers of inpatients suffering from typhoid, paratyphoid fever, and other *Salmonella*-associated infections, including other intestinal infectious diseases, was 629.7 per 100,000 people. Ninety-six deaths due to acute diarrhoea and enteric fever were reported in 2004 (2). This study was conducted as part of the project titled “Institutional cost of shigellosis in Kaengkhoi district, Saraburi province, Thailand.” The project is a part of the multi-country programme sponsored by the International Vaccine Institute, South Korea. Therefore, we would like to focus on bacterial diarrhoea to compare with shigellosis.

The major health-service facilities in Thailand, in terms of the number of settings and beds, are public hospitals consisting of 725 district hospitals, 70 provincial hospitals, and 25 regional hospitals (3). The healthcare system of Thailand comprises three health insurance options: the Social Security Scheme (SSS) for private employees, the Civil Servant Medical Benefit Scheme (CSMBS) for government employees, and the Universal Health Care Scheme (UC) for the remainder of the Thai people. The UC covers approximately two-thirds of the Thai population. The payment methods for hospitals are capitation (a fixed amount per person) for the SSS and the UC and fee-for-service for the CSMBS. The major revenue of the public hospitals comes from capitation. Due to public fiscal constraints and other reasons, the Government has not always approved the capitation rate requested (4). Thus, one of the important management moves in this situation is cost-containment (5). Correspondingly, it is pivotal for hospital administrators to know the cost of treatment, including factors that contribute to this cost, and to use such information for prudent financial management. Hospital cost-analysis and cost-of-illness studies were introduced in Thailand less than a decade ago. And, in fact, a few hospitals have conducted such research. In this light, the present study is aimed at analyzing the health costs of bacterial diarrhoea at a regional hospital (Saraburi Hospital) in central Thailand. Although the study was conducted in one hospital only, it is based on the concept of ‘better than nothing'.

## MATERIALS AND METHODS

This incidence-based cost-of-illness study was conducted from a hospital (provider) perspective, employing a bottom-up or micro-costing approach (6). This study design measures the economic burden on patients from the starting to the end-points of their illnesses. It observes only new cases occurring within a given period and monitors them until reaching the end-point. Since the study sites are public hospitals, the study may be categorized as being from a health system perspective or public perspective.

Cost of illness is composed of direct medical costs, direct non-medical costs, and indirect costs. Direct medical costs are healthcare-related costs directly spent for the prevention, detection, treatment, continuing care, rehabilitation, and terminal care of patients. This study covers only direct medical costs or treatment costs. In a bottom-up approach, costs are collected directly from a patient sample covering individual drug-use and medical services. Medical services include outpatient (routine service) and inpatient services (food, hotel, and routine nursing care), drug-dispensing service, and laboratory investigation service.

One public hospital—Saraburi Hospital—was selected based on the availability of costing information and cooperation of the hospital director and staff. The Saraburi Hospital is a 680-bed, regional tertiary-care hospital in Saraburi province, 108 km northeast of Bangkok. In the study year (2002), the hospital had 1,686 staff members, 1,100 outpatient visits per day, a bed-occupancy rate of 101.36% (informal extra beds were provided), and an average length of hospitalization of 7.14 days.

The study subjects were newly-diagnosed bacterial diarrhoea patients who received treatment at the hospital during 1 October 2000–31 July 2003. The database on medical records of the hospital includes information on 878 outpatient and 233 inpatient cases. Patients were from all age-groups, of both sexes, and constituted both outpatients and inpatients, with a definite diagnosis of diarrhoea classified according to the International Classification of Diseases (10th revision) (7) [Code A00-A05 definitions in Table 1]. Some classifications were based on clinical symptoms. Generally, the diagnosis was based on physical examinations of outpatients and laboratory investigations of inpatients. Patients who were not discharged because of incomplete recovery, or had unimproved conditions in the final treatment, were excluded from the study.

**Table 1.** Demographic and clinical characteristics of patients

Characteristics	Frequency (n=384 episodes)	Percentage
Female/male	216/168	56.3/43.7
Age (years) (mean=23.98 years)		
<5	90	23.4
5-<15	99	25.8
15-55	149	38.8
>55	46	12.0
Type of service		
Inpatient episodes	188	49.0
Outpatient episodes	196	51.0
Payment scheme		
Universal health coverage	130	33.9
Out-of-pocket	117	30.5
Civil Servant Medical Benefit Scheme	97	25.3
Social Security Scheme	40	10.4
ICD code		
A00 Cholera	2	0.5
A01 Typhoid and paratyphoid fevers	2	0.5
A02 Other Salmonella infections	45	11.7
A03 *Shigellosis*	31	8.1
A04 Other bacterial intestinal infections	3	0.8
A05 Other bacterial foodborne intoxications	301	78.4

The sample size for cost-function analysis was based on the rule of at least 30 times the independent or potential predictor variables (8). Considering the factors affecting treatment cost, they were proposed based on a prescribing model. The factors were: health/hospital system, prescriber characteristics, and patient characteristics (9). Since the study was conducted in one hospital, only patient characteristics were explored. From the hypothesis, there were 12 independent variables, including dummy variables, so the minimum sample size was 360 cases. The variables included in the study were categorized into demographic characteristics, i.e. age, gender and health insurance schemes and clinical characteristics, i.e. diagnosis and economic outcome (treatment cost). To collect data, the patients were drawn from the medical database of the hospital, and the medical records were then reviewed. To establish the economic outcomes, the amount of medical services received was multiplied by unit costs, which were, in turn, calculated at the 2002 prices.

The unit costs of medical services were calculated employing a standard costing approach (10,11). This calculation consists of five steps: organizational analysis and cost centre classification, determination of direct cost, determination of indirect cost, determination of full cost, and calculation of the unit cost of medical services (12,13). The departments of the hospital were categorized into 105 patient-service or production cost centres, e.g. laboratory, pharmacy, outpatient, inpatient, and 52 non-patient service or supporting cost centres, e.g. general administration, security, medical statistics, computer centre, and transportation. To qualify as a cost centre, an organizational unit must produce its own output and have a record of resource consumption.

For the determination of direct cost of each cost centre, capital cost was computed as an equivalent annual economic cost (10,14) with a 3% discount rate as per the guidelines of the World Health Organization (15). The equivalent annual economic cost was defined as “an average combination of depreciation cost and interest on the undepreciated portion over the useful life of the capital item” (16). The interest on the undepreciated portion of a capital item was calculated based on the concept of opportunity cost of money spent in advance for that portion. The interest was then calculated for the entire useful life of the capital item and was discounted at the time of analysis. Useful life was considered to be five years and 20 years for capital items and buildings respectively (17,18). Indirect cost, which refers to the direct cost of supporting cost centres, was distributed to the production cost centres by the simultaneous allocation method (10). The simultaneous equation distribution emerges from an attempt to precisely calculate the cost-allocation amounts. By this method, the interdepartmental demands among the general services are more concerned with repeating the assignment of costs among the various cost centres to eliminate the residual costs in the general service departments. These infinite allocations are solved through setting simultaneous linear equations to establish the end of allocation—a method which provides the most accurate result. The services or outputs of supporting cost centres were then selected as allocation criteria, e.g. for the number of staff for the administration department. The average method and micro-costing of departmental allocation were employed for departments producing homogeneous and heterogeneous products respectively (19,20). Micro-costing is a means of allocating the cost of a production cost centre to each unit of service. The micro-costing method begins by valuing resources directly consumed by each unit of service. Then the shared cost of the cost centre is allocated to the various services proportionally to their direct cost.

To analyze the data, descriptive statistics were used for summarizing the study factors. Stepwise multiple regression analysis was employed to formulate the forecasting model of the treatment cost (21). Assumption and model diagnosis were also conducted (21-24). To analyze the uncertainty of results due to the sample data, one-way sensitivity analysis was used in this study. One-way sensitivity analysis is the method of using plausible values of uncertain factors for recalculating the results. The purchasing prices of drugs for hospital were included in sensitivity analysis.

## RESULTS

Table 1 presents the demographic and clinical characteristics of the patients. There were more females (56%) than males. The mean age of the patients was 24 years (median=16.5), and 48% of them were children aged less than 15 years. In terms of payment schemes, 34% of the patients were under the Universal Health Coverage Scheme. Seventy-eight percent of them were classified as ICD code A05 (other bacterial intestinal foodborne intoxications). The unit costs of medical services and drug costs (purchasing prices of the hospital) are presented in Table 2. In total, 384 medical records or episodes (188 outpatients and 196 inpatients) were included in the study. No patient was excluded becauseof unimproved discharge status. We found seven episodes with multiple visits and 17 patients with more than one episode during the study period. For inpatients, the average length of hospitalization was 2.02 days (standard deviation=1.65).

**Table 2.** Unit costs of some medical services and drugs at 2002 prices

Medical service	Unit cost (US$)*
Average routine service in outpatient clinics (per visit)	4.06
Hospitalization; hotel cost with routine nursing care (per day)	
Average of internal medicine wards	18.24
Average of paediatric wards	28.86
Stool examination	0.41
Routine stool culture	5.09
Culture for *V. cholerae*	3.90
Culture for *Shigella* species	3.60
Culture for *Salmonella* species	5.38
Haemoculture	4.53
Anaerobic culture	6.15
Complete blood count	1.61
**Drug**	**Base-case–minimum/maximum prices†**
Norfloxacin 100 mg tablet	1.50-0.75/10.28
Norfloxacin 400 mg tablet	2.45-1.40/5.75
Ciprofloxacin 250 mg tablet	8.80-2.50/3.75
Domperidone tablet	0.78-0.25/1.05
Hyoscine-N-butyl bromide tablet	3.65-1.25/3.40
Metoclopramide 5 mg tablet	0.45-0.35/0.63
Paracetamol 500 mg tablet	0.30-0.20/0.80
ORS adult sachet	6.75-2.50/12.00
ORS paediatric sachet	4.25-2.38/9.00
5% dextrose in half-normal saline solution, 1,000 mL bag	40.00-37.33/63.75
Normal saline solution, 1,000 mL bag	41.25-34.75/149.75

*1 US$=40 Thai bath;

†Price (each) per 100 units; ORS=Oral rehydration solution

As presented in Table 3, treatment cost (direct medical cost) comprised the costs of: outpatient service; hotel, or routine hospitalization service, i.e. room, meals, and routine medical and nursing care; laboratory service; drug acquisition and dispensing; and medical materials. The average treatment cost of all patients (n=384 episodes) was US$ 44.72. Adjusting for the type of care, the average cost per episode of outpatient and inpatient care was US$ 11.29 and US$ 76.78 respectively. The largest portion of these amounts was hotel cost which accounted for US$ 58.82 (77%) per inpatient episode. [These costs can be converted to the 2006 prices using the consumer price index of Thailand (25). Prices increased from a base rate of 100 in 2002 to 114.4 in 2006. Hence, the average treatment cost was US$ 51.16 at the 2006 prices.]

**Table 3.** Average cost per episode (US$ at 2002 prices)

Service	Outpatient episodes	Cost (95% CI)	Inpatient episodes	Cost (95% CI)	Outpatient and inpatient episodes	Cost (95% CI)
Outpatient care	5.25 (4.90-5.60)	N/A	N/A
Inpatient care	N/A	58.82 (51.05-66.60) (average LOS 2.02 days 95% CI 1.79- 2.25)	N/A
Laboratory	0.41 (0.05-0.78)	7.92 (6.75-9.08)	4.24 (3.53-4.98)
Acquisition drug cost	0.86 (0.65-1.08)	3.95 (2.45-5.43)	2.44 (1.65-3.23)
Drug dispensing cost	4.81 (4.48-5.15)	2.27 (2.00-2.55)	3.51 (3.25-3.78)
Medical material cost	0.08 (0.03-0.15)	1.78 (1.48-2.08)	0.95 (0.78-1.13)
Total cost	11.29 (10.38-12.20)	76.78 (66.65-86.90)	44.72 (38.60-50.85)

CL=Confidence interval; LOS=Length of stay; N/A=Not applicable

To test the effect of the variation of drug costs or purchasing prices on the study results, the prices of the top 10 most frequently-used drugs and the top 10 most highly-priced drugs were included. For base-case analysis, the prices were taken from the normal purchasing records of the hospital. The prices for sensitivity analyses were those that were referred from the purchasing reports of hospitals under the Ministry of Public Health in fiscal year 2002 (26). The top 10 most frequently-used drugs were: domperidone tablet; paracetamol 500 mg; hyoscine-N-butyl bromide tablet; norfloxacin 400 mg; ORS (oral rehydration solution) adult; ORS paediatric; norfloxacin 100 mg; metoclopramide 5 mg; 5% dextrose in half-normal saline solution, 1,000 mL; and ciprofloxacin 250 mg. The maximum and minimum prices of drugs were used in recalculating the total drug cost and total treatment cost. The results indicated significant changes in both drug and total treatment costs. When the minimum and maximum prices of drugs were used in recalculation, there were changes of −4.09% to 10.25%, and −0.31% to 0.79% for drug cost and treatment cost respectively. The top 10 most highly-priced drugs used for bacterial diarrhoea were: cefotaxime 1 g; imipenam 500 mg; ceftriazone 1 g; 5% dextrose in half-normal saline solution, 1,000 mL; hyoscine-N-butyl bromide injection; ciprofloxacin 0.1 g/50 mL; ranitidine 50 mg; 5% dextrose in 1/3 normal saline solution, 1,000 mL; cefpirom 1 g; and normal saline solution, 1,000 mL. The maximum and minimum prices of highly-priced drugs affected the total drug cost and the total treatment cost by −31.56% to 29.13% and −2.42% to 2.24% respectively. These effects were greater than those of the top 10 most frequently-used drugs.

To predict the treatment cost, the normal distribution of cost was tested. The results revealed that most costs were not normally distributed (27). Natural logarithms were then applied in the transformation, and further assumption tests and model diagnoses were conducted as guidelines (21,22). The potential explanatory variables included in this analysis were: gender, age-group, health insurance status, and diagnostic group. The result of the multiple regression model is seen in the equation:

Lncost=5.932+1.572(Inpatient)+1.074(A02)+0.203(A03)+0.929(A04)+0.118 (CSMBS)

The adjusted *R*2 of the model was 0.831. This means that 83.1% of the variation in treatment cost could be predicted by the total variables in the model. The predictor variables were composed of: type of patient, i.e. inpatient; type of pathogen, i.e. other *Salmonella* infections [ICD code A02], shigellosis [ICD code A03], or other bacterial intestinal infections [ICD code A04]); and payment (insurance) scheme, i.e. CSMBS).

To estimate the expected response on an untransformed scale after fitting a linear regression model of the transformed scale, it should be adjusted by a smearing factor (28,29). Table 4 shows the predicted treatment cost of patients with various conditions. Patients with health insurance other than the CSMBS, who were infected by other bacterial foodborne intoxications (A05) and who were treated as outpatients, had the lowest cost (US$ 10.27). Meanwhile, patients who were insured by the CSMBS, who had other *Salmonella* infections (A02) and were treated as inpatients, had the highest cost (US$ 162.87).

**Table 4.** Predicted treatment costs at 2002 prices

Type of patients	Predicted treatment cost (US$)
Shigellosis (A03); non-CSMBS outpatient	12.58
Shigellosis (A03); non-CSMBS inpatient	60.58
Shigellosis (A03); CSMBS outpatient	14.15
Shigellosis (A03); CSMBS inpatient	68.17
Other *Salmonella* infections (A02); non-CSMBS outpatient	30.05
Other *Salmonella* infections (A02); non-CSMBS inpatient	144.74
Other *Salmonella* infections (A02); CSMBS outpatient	33.82
Other *Salmonella* infections (A02); CSMBS inpatient	162.87
Other bacterial intestinal infections (A04); non-CSMBS outpatient	26.00
Other bacterial intestinal infections (A04); non-CSMBS inpatient	125.20
Other bacterial intestinal infections (A04); CSMBS outpatient	29.25
Other bacterial intestinal infections (A04); CSMBS inpatient	140.88
Other bacterial foodborne intoxications (A05); non-CSMBS outpatient	10.27

CSMBS=Civil Servant Medical Benefit Scheme

## DISCUSSION

Forty-seven percent of the study patients were aged less than 15 years. The mean age of the patients was 24 years, which was similar to the results of other studies, indicating that diarrhoea is more common in children (30). The hospitalization was relatively short (2.02 days), which can be explained by the fact that the study hospital is a general hospital. There are some chronic and severe patients. Diarrhoea patients are usually admitted to receive intravenous fluid and are then discharged with oral antibiotics. This is done to use hospital-beds efficiently.

Regarding identification of pathogens in terms of ICD code, 90% were identified to be *Salmonella* infections (A04) and other bacterial foodborne intoxications (A05). Patients with specifying pathogens (cholera–0,5%, typhoid–0.5%, and shigellosis–8.1%) were identified based on laboratory investigations at the hospital. This is because most outpatients were diagnosed based on clinical examinations and the experience of physicians. Stool culture takes a few days and, therefore, was not especially useful for the treatment of outpatients. In these situations, physicians provided empiric treatment that was considered to be acceptable (31). It is possible that identification of some pathogens might increase if all cases had been tested by laboratory investigations. Therefore, this study has a limitation in terms of identification of pathogens. In addition, there were no laboratory investigations to confirm the results of treatment of outpatients. We, thus, assumed that the patients had fully recovered. To support this assumption, the data showed that patients would come back to the hospital if they had not recovered as we found several multi-visit episodes.

The average treatment cost of outpatients was relatively high because this study encompassed total economic costs: capital costs, i.e. building, construction, vehicle, equipment, and land; labour costs, such as salary, extra payments, and medical welfare; and material costs. The study also covered both direct cost of patient-care departments and indirect cost allocated from non-patient-care departments. The inpatient cost appeared relatively low compared to that of outpatients, which can be explained based on the length of hospitalization. The average duration of inpatient hospitalization was only 2.02 days. Therefore, hotel cost (the cost of inpatient care) was not especially high, resulting in a low overall treatment cost (since hotel cost is a major part of the treatment cost and since the treatment cost is a result of unit cost of the services multiplied by quantity of the services received). Unit costs of this study are comparable with the hotel cost estimation of the WHO-CHOICE programme (32). The WHO-CHOICE study estimated a cost of 1,000 Thai baht (at the 2000 prices) per patient-day in a tertiary hospital. However, based on this study, the costs are 729 and 1,154 Thai baht at the 2002 prices. At any rate, there is a substantial difference between the costs of outpatient visits estimated by the WHO-CHOICE programme and that of our study (286 Thai baht vs 162 Thai baht respectively).

In testing the uncertainty of results due to costs of drugs, our study demonstrated that the effect of the high-priced drugs was greater than those of the frequently-used drugs. This variation in prices of drugs is similar to that in other countries (33). Therefore, the results of the study indicate that management of drugs in Thailand should not focus solely on frequently-used drugs.

Focusing on the prediction of treatment cost-function, it is generally accepted that outpatient care costs less than inpatient care (34). Therefore, it would be interesting to explore the proportion of inappropriate admissions in further studies. Table 4 presents the difference of costs due to the type of care for identical conditions. The differences in the effects of the various health insurance schemes could be explained by the payer-provider payment methods. The payment method of the primary insurance scheme—CSMBS—was fee-for-service while the other schemes were based on capitation methods. In the fee-for-service schemes, providers tend to offer more services. On the other hand, patients under the SSS incurred lower costs than those under the CSMBS. However, this could be an effect of the health-financing methods. The public hospitals in Thailand receive a fixed per-capita budget (per person registered to the SSS). Physicians are urged to control expenditure with these patients. This phenomenon has been demonstrated in other studies (35-38). Similarly, the effect of insurance schemes on drug costs has been published in the United States (39). Inequity in healthcare should be further explored in this regard.

The model also demonstrated the effect of pathogens on cost. Comparatively, other *Salmonella-*associated infections (ICD code A02) had the highest economic effect. However, the effect of cholera, typhoid, and paratyphoid fever may be inconclusive since a very few cases were found in the present study. The economic effect of pathogens was expected to relate to antimicrobial use and resistance. Antimicrobial resistance is an increasing problem, particularly in developing countries (40). However, we did not include an antimicrobial resistance report of the samples in the study. A 2002 study reported that, in some provinces in southern Thailand, two major serotypes of *Salmonella*—Enteritidis and Typhimurium—were shown to be resistant to tetracycline (57.1% and 83.3% respectively) and cefotaxime (57.1& and 50.0% respectively) (41). In 2001, multidrug-resistant typhoid fever due to *S*. Typhi was investigated in a refugee camp, and 117 of 294 patients showed strong adaptation and remained virulent vis-a-vis streptomycin, ampicillin, chloramphenicol, tetracycline, and sulphamethoxazole-trimethoprim. The rate was only 10% among Thai patients (42).

Hospital administrators should be able to use the results of the present study to achieve more effective management policies. Prices of drugs in Thailand vary greatly. For example, the maximum price of norfloxacin was 13.7 times the minimum price. Similarly, the differences were 4.8 and 3.5 times for oral rehydration solution (ORS) and ciprofloxacin respectively (Table 2). Therefore, costs of drugs could be reduced if locally-made generic drugs were prescribed. In addition, drug-consumption statistics are affected by the use of lower quantities of drugs, but at higher unit prices. This study demonstrated that changes in the prices of the top 10 most frequently-used drugs reduced the costs of drugs by 4% while the top 10 highest-priced drugs raised the costs of drugs by 32%. Regarding service management, while other variables were assumed to be constant, patients under the CSMBS paid a higher cost/treatment rate than patients under other health insurance schemes. This should be a key concern as it violates the principle of equity in healthcare. In terms of infectious disease control, different pathogens resulted in different treatment costs. Investigation of pathogens among patients should, thus, receive more attention, and specific prevention measures and standard practice guidelines should be implemented.

The results of this study may be limited in terms of generalization for the entire country since the data were drawn from only one hospital in Thailand. This is because we experienced various constraints in including more hospitals in the study. Also, since Thailand did not have a standard cost-of-illness menu at the time of the study, we were required to conduct an initial unit-cost analysis for each hospital—a time-consuming and expensive endeavour. Although the results of this study may not be applicable to other hospitals, they could, however, provide cost-conscious ideas to the Thai health ministry and to both practitioners and administrators of Thai hospitals. Lastly, others can use the results of the study as a basis for further research.

In conclusion, the study found that nearly half of the patients with bacterial diarrhoea in Thailand were aged less than 15 years. Approximately half of them were treated as inpatients. The average estimated treatment cost, including cost-predicting variables, was US$ 45 per episode. Likewise, the effect of prices of drugs on treatment was explored. The types of service (inpatient or outpatient), pathogens, and health-insurance schemes significantly affected the predicted cost. The fitted model was able to predict greater than 80% of the treatment cost. The estimation of simulated patients demonstrated a wide range of costs, from US$ 10 per episode to US$ 163 per episode. Overall, hospital administrators can apply these results in cost-containment interventions.

## ACKNOWLEDGEMENTS

The authors thank the sponsor—the Faculty of Graduate Studies of Mahidol University, Bangkok, Thailand—for the contract grant. The authors also thank Mr. Perry Whalley of the Language Centre, Faculty of Graduate Studies of Mahidol University, for his advice on presentation of the manuscript.
